# Emerging Role of m^6^ A Methylome in Brain Development: Implications for Neurological Disorders and Potential Treatment

**DOI:** 10.3389/fcell.2021.656849

**Published:** 2021-05-19

**Authors:** Godwin Sokpor, Yuanbin Xie, Huu P. Nguyen, Tran Tuoc

**Affiliations:** ^1^Department of Human Genetics, Ruhr University of Bochum, Bochum, Germany; ^2^Department of Biochemistry and Molecular Biology, Gannan Medical University, Ganzhou, China

**Keywords:** mRNA methylation, mRNA metabolism, N^6^-methyladenosine (m^6^A), cortical development, neurological disorders, clustered regularly interspaced short palindromic repeats (CRISPR)–dCas13b, m^6^A editing

## Abstract

Dynamic modification of RNA affords proximal regulation of gene expression triggered by non-genomic or environmental changes. One such epitranscriptomic alteration in RNA metabolism is the installation of a methyl group on adenosine [N^6^-methyladenosine (m^6^A)] known to be the most prevalent modified state of messenger RNA (mRNA) in the mammalian cell. The methylation machinery responsible for the dynamic deposition and recognition of m^6^A on mRNA is composed of subunits that play specific roles, including reading, writing, and erasing of m^6^A marks on mRNA to influence gene expression. As a result, peculiar cellular perturbations have been linked to dysregulation of components of the mRNA methylation machinery or its cofactors. It is increasingly clear that neural tissues/cells, especially in the brain, make the most of m^6^A modification in maintaining normal morphology and function. Neurons in particular display dynamic distribution of m^6^A marks during development and in adulthood. Interestingly, such dynamic m^6^A patterns are responsive to external cues and experience. Specific disturbances in the neural m^6^A landscape lead to anomalous phenotypes, including aberrant stem/progenitor cell proliferation and differentiation, defective cell fate choices, and abnormal synaptogenesis. Such m^6^A-linked neural perturbations may singularly or together have implications for syndromic or non-syndromic neurological diseases, given that most RNAs in the brain are enriched with m^6^A tags. Here, we review the current perspectives on the m^6^A machinery and function, its role in brain development and possible association with brain disorders, and the prospects of applying the clustered regularly interspaced short palindromic repeats (CRISPR)–dCas13b system to obviate m^6^A-related neurological anomalies.

## Introduction

Over 170 chemical modifications of RNA are known to exist in eukaryotes (Boccaletto et al., 2018). These RNA modifications, together referred to as the epitranscriptome, play essential roles in gene expression regulation *via* affecting RNA metabolism: RNA processing, decay, transport, and translation. N^6^-methyladenosine (m^6^A) is among the characterized adenosine methylations of messenger RNAs (mRNAs) (Engel and Chen, 2018) and the most occurring in mammalian cells (Roundtree et al., 2017a). The m^6^A mRNA methylome is dynamically regulated by factors that install, remove, or bind the m^6^A mark on mRNA. Such dynamism in the m^6^A landscape is known to critically regulate mRNA metabolism to influence gene expression. In essence, m^6^A modification is reported to modulate several biological events, including cell proliferation, differentiation, and embryonic development, and can also lead to disease conditions when dysregulated (Dominissini et al., 2012; Meyer et al., 2012; Ke et al., 2015; Linder et al., 2015; Zhao et al., 2017; Ries et al., 2019).

The impact of m^6^A modification on cell biological processes is notable in nervous tissues (Widagdo et al., 2016; Li et al., 2019). This is because neural cells are known to be enriched with m^6^A-tagged mRNAs. As a result, the developing and adult brain is reported to be ubiquitously enriched with m^6^A modifications (Chang et al., 2017; Zhang F. et al., 2018). The m^6^A level in the brain is temporally regulated in the course of its development such that the adult brain registers the highest level of m^6^A (Meyer et al., 2012). The massive prevalence of m^6^A in the developing and postnatal brain signifies the importance of m^6^A modification in regulating brain morphogenesis and function. Indeed, a chunk of the expanding knowledge indicating the phenomenal role of m^6^A in orchestrating neural development and function includes the proliferation of neural stem cells (NSCs) and other neural precursor cells, neuroprogenitor cell differentiation, gliogenesis, elaboration of neural processes, and synaptic transmission (reviewed in Widagdo and Anggono, 2018; Li et al., 2019; Chokkalla et al., 2020; Dermentzaki and Lotti, 2020; Livneh et al., 2020).

While maintenance of the general m^6^A homeostasis is indispensable for proper brain development and activity, selective hypomethylation and hypermethylation of gene transcripts are critical mechanistic modalities for normal brain neurodevelopment and functionality. Moreover, the targeted binding of m^6^A on transcription factor-encoding mRNAs and disease-risk gene transcripts in the developing brain (Zhang et al., 2020) reflects how m^6^A signaling is critical for brain structure and function in health. It also means that disturbance of the m^6^A RNA methylome can have implications for abnormal anatomy and physiology of the brain, leading to neurological disorders.

In this review, we present the role of the m^6^A methylation machinery in mRNA metabolism, with discussion focused on how the m^6^A landscape regulates brain development and function. Neurodevelopmental, neurodegenerative, and neuropsychiatric disorders of the brain attributable to m^6^A signaling dysregulation are highlighted.

## The N^6^-Methyladenosine Modification Machinery

**FIGURE 1:**
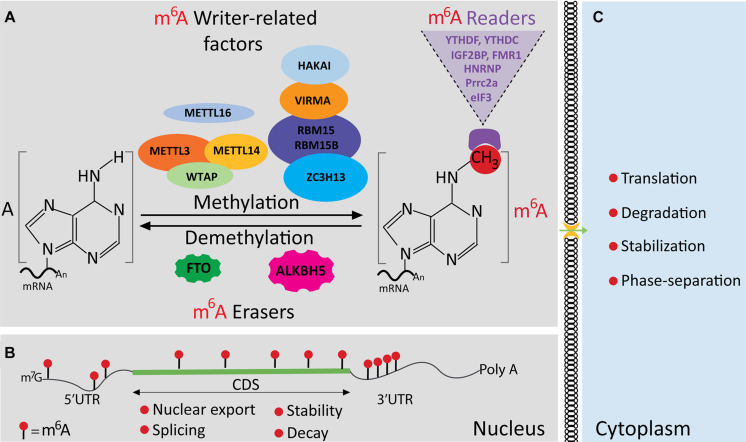
The N^6^-methyladenosine (m^6^A) machinery and modification of messenger RNA (mRNA). **(A)** An illustration showing the m^6^A machinery. It is made of factors that can functionally be categorized as writers, erasers, and readers of m^6^A. The m^6^A marks are deposited by the methylation complex (writers), including METTL3, METTL14, and WTAP, which is counteracted by the m^6^A demethylases (FTO and ALKBH5), leading to m^6^A removal. The recognition of m^6^A marks is done by the readers or binding proteins as indicated. **(B)** Diagram showing a typical m^6^A distribution in regions of an mRNA located in the nucleus. **(C)** The m^6^A readout affects mRNA fates, including trafficking, stability, decay, translation, and localization.

Early studies revealed that m^6^A is the most substantial posttranscriptional modification in eukaryotic mRNAs (Desrosiers et al., 1974; Perry et al., 1975a, b). It was proposed that nearly 7,000 mRNAs from human and mouse transcriptome contain m^6^A modification (Dominissini et al., 2012; Meyer et al., 2012). Currently, over 10,000 m^6^A-modified mRNA transcripts have been identified in yeast and mammalian cells (Wang and Zhao, 2016). m^6^A modification and recognition (binding) are achieved by three functional components of the m^6^A machinery, namely, m^6^A methyltransferases (“writers”), m^6^A demethylases (“erasers”), and m^6^A binding or interacting proteins (“readers”) (Figure 1A). Through the specific binding of m^6^A-interacting proteins, m^6^A mRNA methylation is able to play a central role in regulating several aspects of mRNA metabolism, such as transport, splicing, stability, translation, and phase separation (Figure 1). High-throughput data revealed that m^6^A modification typically occurs within the consensus sequence RRACH (R stands for G or A; H stands for A, C, or U) (Dominissini et al., 2012; Meyer et al., 2012). The consensus sequence was recently redefined as DRACH, where D stands for G, A, or U (Linder et al., 2015). As depicted in Figure 1B, m^6^A distribution along mRNA is asymmetric. In general, m^6^A sites are concentrated in the protein coding region (CDS) near stop codons, followed by the 3′ untranslated region (UTR), and in the 5′ UTR (Figure 1B) (Dominissini et al., 2012; Meyer et al., 2012; Ke et al., 2015; Linder et al., 2015).

### N^6^-Methyladenosine Writers

The installation of m^6^A is carried out by ∼1 MDa m^6^A writer complex composed of the methyltransferase-like protein 3 (METTL3) and METTL14, which heterodimerize (METTL3-METTL14) to function as the enzymatic core of the writer complex (Bokar et al., 1994; Bujnicki et al., 2002; Liu et al., 2014; Iyer et al., 2016). Additionally, other factors are known to interact with the m^6^A writer complex. These include Wilms tumor 1-associated protein (WTAP) (Ping et al., 2014), VIRMA/KIA1429 (Yue et al., 2018), RNA-binding protein 15 (RBM15) (Patil et al., 2016; Huang and Yin, 2018), ZC3H13 (Knuckles et al., 2018), and HAKAI (Yue et al., 2018) (Figure 1A). These cofactors are regulated by the binding of RNA and the catalytic activity of the enzymatic core of the m^6^A writer complex (Bujnicki et al., 2002; Liu et al., 2014; Ping et al., 2014; Iyer et al., 2016; Yue et al., 2018).

#### METTL3 and METTL14

Bokar et al. (1994) partially purified the m^6^A writer complex using an *in vitro* methylation system and identified MT-70, a 70-kDa sub-complex possessing S-adenosylmethionine-binding methyltransferase capacity (Bokar et al., 1994). Later, it was renamed METTL3 (Narayan and Rottman, 1988; Bokar et al., 1997). Knockout of METTL3 in cells effectively blocks m^6^A modification of mRNAs (Zhong et al., 2008; Agarwala et al., 2012; Geula et al., 2015). On the other hand, METTL14 forms a stable heterodimer with METTL3 to form the methyltransferase core of the m^6^A methylation machinery (Liu et al., 2014; Wang Y. et al., 2014). METTL14, however, lacks enzymatic function and instead acts as an RNA-binding scaffold to augment the enzyme activity of METTL3 by directing the location of SAM methyl group required for the reaction (Śledź and Jinek, 2016; Wang P. et al., 2016; Wang X. et al., 2016). Therefore, METTL3 is the primary enzyme responsible for m^6^A installation on mRNA.

#### METTL3-METTL14-Associated Adaptors: WTAP, VIRMA (KIAA1429), RBM15/15B, ZC3H13 (KIAA0853), and HAKAI

The core m^6^A writer complex METTL3-METTL4 interacts with other adaptor proteins. It was found that FIP37 (the plant homolog of WTAP) co-localized with MTA (*Arabidopsis* homolog of METTL3) in the nucleus through physical interaction (Zhong et al., 2008). Similar interaction between WTAP and METTL3 was observed in mammalian cells (Liu et al., 2014; Ping et al., 2014; Schwartz et al., 2014). WTAP is key in keeping the METTL3-METTL4 heterodimer in nuclear speckles (Ping et al., 2014). Loss of WTAP leads to the depletion of m^6^A modification in mRNA, indicating that WTAP may orient METTL3-METTL14 onto targets (Ping et al., 2014). However, the detailed mechanism remains elusive. Of note, it was demonstrated that two classes of m^6^A sites exist: WTAP-dependent and WTAP-independent sites (Schwartz et al., 2014). VIRMA is known to also interact with the WTAP-METTL3-METTL4 complex (Figure 1A; Schwartz et al., 2014) and indicates its essentiality for the m^6^A writer complex functionality. Indeed, VIRMA deletion in human cells leads to a significant reduction in mRNA methylation levels, although not as intense as that resulting from WTAP knockdown (Schwartz et al., 2014). Biochemical studies from Yue et al. (2018) demonstrated that VIRMA preferentially regulates m^6^A modification in the 3’ UTR proximal to stop codon.

Through proteomic studies, Horiuchi et al. (2013) observed that RBM15 and its paralog RBM15B, together with ZC3H13/KIAA0853, and MTA70 (METTL3), associate with WTAP (Horiuchi et al., 2013; Patil et al., 2016), which raises the possibility that RBM15 and RBM15B may also play role(s) in m^6^A modification. Indeed, silencing of RBM15 and RBM15B led to a demonstrable decrease in m^6^A levels of mRNA (Patil et al., 2016). Based on Individual-nucleotide resolution UV crosslinking and immunoprecipitation (iCLIP) data, it was proposed that RBM15/15B recruit the m^6^A methylation machinery to perform m^6^A modification through binding to uridine-rich regions near DRACH sites. That notwithstanding, it is not always the case that uridine-rich regions exist near m^6^A sites; therefore, other methylation complex adaptors may mediate the complex binding to such variant m^6^A sites (Patil et al., 2016; Meyer and Jaffrey, 2017).

ZC3H13/KIAA0853 is also an interactor of the m^6^A machinery, and it is demonstrated to be crucial in linking RBM15/15B to WTAP (Horiuchi et al., 2013; Knuckles et al., 2018; Wen et al., 2018). Knockdown of ZC3H13 shifts the localization of the m^6^A adaptors WTAP, Virilizer, and Hakai from nucleus to cytosol in embryonic stem cells and leads to a significant total reduction in m^6^A level on mRNA (Wen et al., 2018). This reflects an essential role of ZC3H13 in the deposition of m^6^A on mRNAs. The E3 ubiquitin ligase HAKAI (CBLL1) is another notable factor that interacts with the m^6^A machinery (Figure 1A; Horiuchi et al., 2013; Rùžièka et al., 2017). However, its function in m^6^A modification of mRNA in mammals is yet to be established.

### Erasers (Demethylases) of N^6^-Methyladenosine

m^6^A modification is believed to be a reversible dynamic process premised on the identification of two demethylases: fat mass and obesity-associated protein (FTO) (Jia et al., 2011) and α-ketoglutarate-dependent dioxygenase alkB homolog 5 (ALKBH5) (Figure 1A). However, this important concept has been in controversy due to various supporting data from various studies (Mauer et al., 2017; Darnell et al., 2018; Wei et al., 2018) as discussed below.

#### Fat Mass and Obesity-Associated Protein

Following an *in vitro* assay, which demonstrated that FTO erases m^6^A methylation of mRNA (Jia et al., 2008), it was further shown that downregulation (knockdown) of *FTO* in HeLa or 293FT cells caused reduction in m^6^A methylation of mRNA (Jia et al., 2011). In support of this observation, it was identified that a small proportion of m^6^A peaks of the whole transcriptome increased in *Fto* knockout mouse (Hess et al., 2013). These evidence consolidates the concept that m^6^A modification can be reversed by FTO functionality. However, this idea was challenged by another study, in which no significant increase in m^6^A level was observed in *Fto* knockout cells (Mauer et al., 2017). Instead, they noticed that FTO exhibits much higher catalytic capacity against m^6^Am than m^6^A. These studies indicate that the preferred substrate of FTO may be m^6^Am (Meyer and Jaffrey, 2017). What could be the explanation behind the discrepancy between these findings? It is worth pointing out that several independent groups have reported that the Kcat/Km of FTO against m^6^A is in the range of 0.6−0.7 min^–1^ μM^–1^ (Jia et al., 2011; Zhu and Yi, 2014; Zou et al., 2016), whereas that from the study of Mauer et al. (2017) is only 0.06 min^–1^ μM^–1^, indicating that most likely there is a technique issue behind quantification of the Kcat/Km of FTO against m^6^A. Additionally, both investigations used different methods to determine the level of m^6^A, noting that the RNase T1 treatment of mRNA combined with thin-layer chromatography can only measure the m^6^A in the case of RGACH, but not RAACH (Mauer et al., 2017). Moreover, a recent study further demonstrates that FTO not only demethylates internal m^6^A but also caps m^6^Am (Wei et al., 2018). The subcellular distribution of FTO varies among cultured cell lines, which indicates that the pattern of FTO demethylation of m^6^A in cytosol or nucleus could be cell lineage-dependent. Consistent with the above studies, it was found that FTO plays a vital role in cell cycle and mitosis regulation in an m^6^A demethylation-dependent manner during spermatogenesis (Huang T. et al., 2018).

Structural studies uncovered that FTO prefers m^6^A-modified nucleobase, and its demethylase activity can be influenced by the primary and the tertiary structure of target RNA (Zhang X. et al., 2019), thus shedding light on the molecular mechanism behind the demethylation function of FTO. Recent findings show that the transcription of FTO is regulated by a transcriptional factor Zfp217 during adipogenesis, and Zfp217 is critical for FTO to associate with m^6^A sites, albeit through competition with YTHDF2 for binding sites (Wei et al., 2019).

#### ALKBH5

ALKBH5 is another m^6^A factor with demethylase capacity (Jia et al., 2011; Zheng et al., 2013). Manipulating ALKBH5 expression level leads to a slight but significant change in m^6^A levels in the poly(A) region of mRNA. Compared with FTO, which demethylates m^6^Am and m^6^A, ALKBH5 shows specificity for m^6^A demethylation (Wei et al., 2018). Importantly, m^6^A-mediated conformational change facilitates distinction of substrates with minor sequence by FTO and ALKBH5 (Zou et al., 2016). As a nuclear protein, ALKBH5 is proposed to only erase the m^6^A methylation in the nucleus (Meyer and Jaffrey, 2017). The demethylation capacity of ALKBH5 plays important roles in mRNA splicing, transport, stability, and processing. For instance, spermatogenic transcripts with increased m^6^A levels exhibit increased splicing events in *Alkbh5* KO mice (Tang et al., 2018). Recently, it was reported that METTL3 and ALKBH5 counteractively modulate the m^6^A methylation of TFEB transcript to effect regulation of autophagy (Song et al., 2019). The demethylation activity of ALKBH5 can be regulated by DEAD-Box RNA helicase through physical interaction (Shah et al., 2017).

### Readers (Binding Proteins) of N^6^-Methyladenosine

The functional significance of m^6^A modification also depends on m^6^A-binding proteins also referred to as m^6^A readers. As described below, we categorize the readers in mammalian cells into two groups: YTH domain-containing proteins, including YTHDC1, YTHDC2, and YTHDF1−3 (Hazra et al., 2019), and Non YTH domain-containing proteins, including eIF3 (Meyer et al., 2015), IGF2BPs (Huang H. et al., 2018), HuR (Dominissini et al., 2012; Wang Y. et al., 2014), FMRP (Zhang F. et al., 2018), hnRNPA2/B1/C (Dominissini et al., 2012; Alarcón et al., 2015), and METTLs (Wang et al., 2015).

#### YTH Domain Containing N^6^-Methyladenosine-Binding Proteins

This group contains YTHDC1, YTHDC2, and YTHDF1−3 families in mammals. The common YTH domain defines members of this group of m^6^A binding and determines the nature of m^6^A reading (Zhang et al., 2010). However, they are not paralogs. This is because of the non-similarity of other aspects of proteins apart from the common YTH domain (Hazra et al., 2019).

**FIGURE 2:**
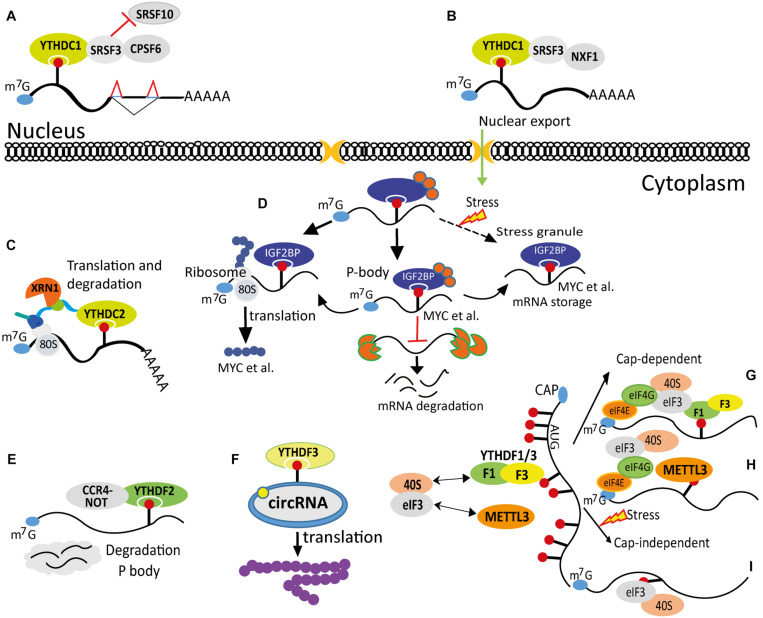
Effects of N^6^-methyladenosine (m^6^A) methylation on messenger RNA (mRNA) fate. **(A)** m^6^A modification regulates mRNA splicing and polyadenylation *via* YTHDC1 and its associating factors SRSF3, SRSF10, and CPSF6. **(B)** m^6^A modulates mRNA nuclear export through YTHDC1, SRSF3, and NXF1. **(C)** m^6^A regulates mRNA translation and stability *via* YTHDC2-mediated recruitment of the ribosome and the XRN1 exoribonuclease. **(D)** m^6^A marks are bound by IGFBPs, which can regulate a subset of mRNA translation, decay in P-body, and storage in stress granules. **(E)** m^6^A modification regulates mRNA degradation in P-body through associating with the YTHDF2-CCR4-NOT complex. **(F)** m^6^A marks on circRNA modulate its translation *via* recruiting YTHDF3. **(G)** m^6^A marks recruit YTHDF1/YTHDF3 to enhance translation in a Cap-dependent manner. **(H)** METTL3 serves as an m^6^A reader and increases translation *via* recruiting translation initiation complex independent of its methyltransferase activity. **(I)** m^6^A directly binds to eIF3 and increases translation in a Cap-independent manner.

YTHDC1 (YT521-B) is the first identified m^6^A reader, which was found as a protein associated with splicing factors (Imai et al., 1998; Harfmann et al., 1999; Xiao et al., 2016; Hazra et al., 2019). Interestingly, human YTHDC1 shows much greater binding affinity for the m^6^A-modified mRNA region in the context of Gm^6^AC (five-fold to six-fold difference) than Am^6^AC, although the distribution of m^6^A modification found in the consensus sequence is Gm^6^AC (70%) and Am^6^AC (30%) (Xu et al., 2014, 2015). It localizes in various subnuclear bodies close to the nuclear splicing factor (SF) compartments and plays a role in mRNA splicing through physical interaction with splicing factor SRSF3 and SRSF10 (Figure 2A; Xiao et al., 2016). Furthermore, YTHDC1 works together with NXF1 and SRSF3 to regulate m^6^A-modified mRNA nuclear export (Figure 2B; Roundtree et al., 2017b). Moreover, YTHDC1 has been reported to bind m^6^A-modified MAT2A mRNA. The m^6^A modification results in the degradation of MAT2A mRNA, although the detailed mechanism is not known (Shima et al., 2017).

On the other hand, YTHDC2 is a multi-domain protein and mainly localized in the cytoplasm, but it is also highly expressed in perinuclear compartment. It prefers to bind m^6^A-containing RNAs through the YTH domain and enhances RNA degradation. Meanwhile, it also enhances m^6^A-modified mRNA translation efficiency (Kretschmer et al., 2018).

Human YTHDF1−3 proteins contain a YTH domain in the C-terminus and a low-complexity domain in the N-terminus. These three members of the YTHDF family share high sequence identity and similarity (65%−80%) (Li et al., 2014; Wang X. et al., 2014; Hazra et al., 2019). As a characterized m^6^A modification reader, the human YTHDF2 binds over 3,000 transcripts primarily in their 3′ UTRs and around the stop codon. The binding of YTHDF2 leads to degradation of the bound mRNAs in cytoplasmic processing bodies (P-bodies). Knockdown of YTHDF2 leads to an accumulation of m^6^A-containing mRNAs (Wang X. et al., 2014). YTHDF2 was also found to associate with CNOT1, the scaffolding component of the CCR4-NOT mRNA deadenylation complex (Figure 2E). This interaction is required for YTHDF2 to localize in P-bodies (Du et al., 2016). Therefore, the main function of YTHDF2 is to control the degradation of m^6^A-modified mRNAs (Kang et al., 2014; Hazra et al., 2019).

Unlike YTHDF2, YTHDF1 does not induce the degradation of associated m^6^A-containing mRNAs. Instead, but arguably, YTHDF1 increases the translation efficiency of associated mRNAs (about 1,200) in an m^6^A-dependent fashion (Wang et al., 2015). This function of YTHDF1 is further supported by the work of Wu et al. (2019), who showed that YTHDF1 targets m^6^A-modified *Jak2* and regulates its translation (Wu et al., 2019). Recently, another cytoplasmic m^6^A reader protein YTHDF3 was found to interact with YTHDF1 to promote translation, whereas YTHDF3 interacts with YTHDF2 to reinforce mRNA decay (Li A. et al., 2017; Shi et al., 2017). Furthermore, biochemical studies showed that YTHDF3 shares greater than 50% of common m^6^A-modified mRNA targets with YTHDF1 and also with YTHDF2 (Li A. et al., 2017; Shi et al., 2017). In addition, YTHDF3 might also function as m^6^A-modification reader independent of YTHDF1 and YTHDF2 under certain conditions. Oxidative stress induces specific m^6^A modifications in a set of transcripts, and the binding of YTHDF3 to the modifications triggers the mRNA–YTHDF3 complex localization in the stress granules, but without much influence on YTHDF1 and YTHDF2 (Anders et al., 2018). Importantly, YTHDF3 can also enhance translation independent of METTL3-mediated m^6^A modification. For example, YTHDF3 functions together with eIF4G2 and Poly(A)-binding protein 1 (PABP1) to promote the translation of forkhead box protein O3 (FOXO3) (Zhang Y. et al., 2019).

Interestingly, very recent studies have shown evidence indicating functional redundancy of the YTHDFs during mRNA degradation and cellular differentiation. As such, it is only when all three YTHDF homologs (YTHDF1−3) are ablated that mRNA stability and cell differentiation regulation become evident (Kontur et al., 2020; Zaccara and Jaffrey, 2020). This may partly stem from the observations that all three YTHDFs are similar in sequence characteristics and usually have common mRNA binding targets (Zaccara and Jaffrey, 2020). Yet, it has been reported that probably due to variation in its expression, YTHDF2 dominates the m^6^A reader function of all the YTHDFs (Lasman et al., 2020). It was also unraveled that YTHDFs are unable to induce translation in HeLa cells (Zaccara and Jaffrey, 2020). While these new findings present a unified model seeking to define the regulatory functions of YTHDFs in m^6^A modification, they provoke questions that need to be addressed to reconcile the discrepancy between the recent findings and previous observations with respect to the precise role of YTHDFs in mRNA translation.

#### Non YTH Domain Containing N^6^-Methyladenosine Readers (eIF3, IGF2BPs, HuR, FMRP, HNRNP Proteins, and PRRC2A)

Meyer et al. (2015) characterized the function of eIF3 as an m^6^A reader. eIF3 is preferentially recruited by the m^6^A-modified mRNA over unmethylated mRNA (Meyer et al., 2015). It was shown that about 35% of m^6^A marks in the 5′ UTR are also eIF3-binding sites. Depletion of m^6^A through METTL3 loss-of-function decreased the translation of m^6^A-modified mRNA in the 5′ UTR, but not the mRNAs bearing m^6^A marks elsewhere (Meyer et al., 2015). Notably, one of the two modes of m^6^A-mediated Cap-independent translation is through direct association of m^6^A in the 5′ UTR and eIF3 (Figure 2I; Meyer et al., 2015), while the other mode involves YTHDF1 association with m^6^A mark followed by delivery of eIF3 to the 5′ UTR (Figure 2G; Wang et al., 2015). This indicates the correlation between the 5′ UTR m^6^A and translation and highlights the involvement of eIF3 in the regulation of mRNA translation. Currently, it is not known what the detailed mechanisms are in determining the mode of eIF3−5′ UTR association.

Insulin-like growth factor-2-binding proteins (IGFBPs), including IGFBP1−3, have been reported as RNA-binding proteins (Bell et al., 2013). Recently, it was demonstrated that IGFBP1−3 bind m^6^A-modified mRNAs with a three-fold to four-fold greater affinity than the m^6^A-unmodified mRNAs (Huang H. et al., 2018). By means of RIP-Seq or PAR-CLIP-Seq, it was found that IGFBP1−3 share 55%−70% RNA targets with preference for binding to the “UGGAC” consensus motif, e.g., *MYC*, *FSCN1*, and *TK1* (Huang H. et al., 2018). Knockdown of METTL14, a critical component of the methylation machinery, dramatically undermined IGFBP binding. Interestingly, knockdown of IGF2BPs reduces mRNA stability (Huang H. et al., 2018). Consistently, IGFBPs were found to associate with three mRNA stabilizing factors, including HuR, MATR3, and PABP1, which can support IGFBPs in stabilizing their mRNA targets (Huang H. et al., 2018).

HuR is an RNA-binding protein with multiple molecular functions. It was first described as a stabilizer of ARE-containing mRNAs (Fan and Steitz, 1998; Peng et al., 1998). It is also known to enhance translation, although it can also exert translation suppression (Hinman and Lou, 2008; Abdelmohsen and Gorospe, 2010). This portrays HuR as both a reader and anti-reader of m^6^A (Dominissini et al., 2012; Wang Y. et al., 2014). However, the underlying mechanism that makes m^6^A modification sites to recruit or block HuR binding is unknown. We think that a sequence-dependent context may be at play in determining the function of HuR in m^6^A interaction. This speculation remains to be investigated.

FMR1 (also known as FMRP1) is an RNA-binding protein and known to associate with hundreds of transcripts to decrease their translation. It binds to m^6^A-modified mRNA in an RNA sequence context-dependent manner. FMR1 selectively binds to the m^6^A marks associated with GGACU RNA sequence (Edupuganti et al., 2017). Bioinformatic analysis revealed that FMR1 and YTHDF1 shared an abundant set of common m^6^A-modified mRNAs, indicating that FMR1 might compete with YTHDF1 for binding of m^6^A-modified mRNAs to downregulate translation (Ascano et al., 2012; Wang et al., 2015). It is possible that the mechanism may underlie the previously reported regulatory function on the translation of mRNA targets.

Heterogeneous nuclear ribonucleoproteins (hnRNPs: hnRNPA2/B1, hnRNPC, and hnRNPG) are RNA-binding proteins that play important roles in pre-RNA processing (Dominissini et al., 2012; Alarcón et al., 2015; Liu et al., 2015, 2017; Xiao et al., 2016). Alarcón et al. (2015) discovered that hnRNPA2B1 interacts with a group of m^6^A-modified RNAs in the nucleus and regulates their splicing in a comparable pattern as for METTL3. However, the binding of hnRNPA2/B1 to m^6^A is likely indirect and may require an hnRNPC-mediated switch mechanism to do so (Wu et al., 2018). hnRNPC can read m^6^A-modified hairpin and m^6^A-containing RNAs. m^6^A-modification leads to a change in the regional RNA structure and increases the binding of hnRNPC (Liu et al., 2015). Consistently, general reduction in m^6^A marks due to METTL3/L14 knockdown eliminates the association of hnRNPC to the aforementioned m^6^A-mediated RNA structural modification (Liu et al., 2015). Furthermore, HNRNPG is known to bind m^6^A-modified lncRNA through its C-terminal low-complexity domain (LCD), indicating that LCD domain might be used by some other readers to bind to m^6^A modification (Liu et al., 2017).

Recently, PRRC2a was reported as an m^6^A modification reader (Wu et al., 2019). Through RIP-seq and m^6^A-seq, it was identified that PRRC2a binding peaks within over 2,800 genes in brain samples, and PRRC2a mainly binds to the consensus motif UGGAC in m^6^A-modified transcripts (Wu et al., 2019). PRRC2A was found to be associated with YTHDF2 in granule-like organelles, which may be involved in the regulation of PRRC2A involvement in *Olig2* mRNA stability (Wu et al., 2019). However, since PRRC2A has low tissue expression specificity, it is unclear whether PRRC2A serves as an m^6^A modification reader in other tissues.

#### Reader Function of METTLs

Besides its role as an m^6^A writer, METTL3 can also bind to m^6^A-modified mRNAs to act as a reader. It was found that METTL3 regulates the translation of some oncogenic m^6^A-modified mRNAs independent of its methyltransferase activity through eIF3 recruitment to the translation initiation complex (Lin et al., 2016). A study from the same group identified a physical interaction between m^6^A-bound METTL3 near the stop codon and eIF3h, providing a mechanism to explain how METTL3 can enhance translation (Choe et al., 2018). The methyltransferase METTL16 also serves as an m^6^A reader in a certain context. When SAM concentrations become low, METTL16 remains bound to m^6^A-modified MAT2A in its 3′ UTR hp1 site to enhance MAT2A splicing, resulting in increased MAT2A levels in the cytosol. On the contrary, when SAM levels are high, METTL16 methylates MAT2A and facilitates intron retention (Pendleton et al., 2017).

### Deposition of N^6^-Methyladenosine Modification During Transcription

Mechanistically, how m^6^A modification of transcripts is carried out needs elucidation. A recent study uncovered an insightful detail in the installation of m^6^A. Specifically, it was found that H3K36me3 cooperates with METTL3/METTL14 to deposit m^6^A on mRNA (Huang et al., 2019). The study showed that H3K36me3 physically interacts with METT14, thus recruits the m^6^A methylation machinery to RNA Pol II, and allows the m^6^A methylation machinery to effect m^6^A modification during transcription. Decreasing the level of H3K36me3 through loss-of-function of SETD2, the specific enzyme that converts H3K36me2 or H3K36me0 to H3K36me3, significantly led to the reduction in m^6^A level on RNAs, mimicking the impact of depletion of individual m^6^A writer complex components (Huang et al., 2019).

### Impact of N^6^-Methyladenosine Modification on Gene Regulation

The reversible modification of m^6^A exerts functional impact on several aspects of mRNA metabolism, including nuclear export, polyadenylation, splicing, degradation, and translation (Figure 2). By these means, the m^6^A methylome affords an additional level of gene expression regulation to sculpt the transcriptome (Fu et al., 2014).

#### N^6^-Methyladenosine Modification Regulates mRNA Splicing

Some factors involved in m^6^A modification of mRNA are known to interact with pre-mRNA splicing factors (SRSFs), indicating a possible role for m^6^A in mRNA splicing (Zhao et al., 2014; Xiao et al., 2016). It has been demonstrated that enrichment of m^6^A modification promotes recruitment of SRSF2 and leads to enhanced exon inclusion of target mRNA (Zhao et al., 2014). It has been further suggested that the m^6^A reader YTHDC1 regulates the association of m^6^A and SRSFs. Indeed, m^6^A-bound YTHDC1 enhances the recruitment of SRSF3 that favors exon inclusion but blocks the recruitment of SRSF10, an exon skipping-related splicing factor (Xiao et al., 2016). Moreover, hnRNPs may also be involved in the regulation of RNA splicing (Liu et al., 2015, 2017). For example, the modification of m^6^A on pre-mRNA favors the binding of hnRNPC (Liu et al., 2015), which could further facilitate splicing through its known function in repressing exon inclusion (Zarnack et al., 2013). Therefore, it is possible that perturbation of the m^6^A machinery components can impair mRNA alternative splicing. This idea is especially supported by the observation that knockdown of METTL3 can antagonize the association of SRSF2 or SRSF3 with m^6^A-modified pre-mRNAs (Zhao et al., 2014; Xiao et al., 2016), and facilitates the expression of the long isoform of MyD88 (MyD88L) *via* exon skipping attenuation (Feng et al., 2018). Additional evidence is also based on the essential role played by METTL16 in MAT2A-mediated pre-mRNA alternative splicing (Pendleton et al., 2017).

#### N^6^-Methyladenosine Controls Alternative Polyadenylation

Ke et al. (2015) found that m^6^A modification peaks in the 3′ UTR, especially for transcripts that use alternative polyadenylation (APA), and longer last exons exhibit a higher m^6^A density. By comparing the m^6^A density of thousands of mRNA UTRs from liver and brain tissues, it was observed that greater amount m^6^A marks in the last exons are linked to the usage of more distal polyA sites. Indeed, global reduction of m^6^A levels *via* triple knockdown of METTL3, METTL14, and WTAP changed the polyA sites of one-sixth of the examined 661 mRNAs and promoted the usage of proximal APA sites, indicating that some m^6^A marks inhibit proximal polyadenylation (Ke et al., 2015).

Recently, a mechanism through which m^6^A controls alternative polyadenylation was proposed. VIRMA (Figure 1A) was found to interact with polyA cleavage factors F5 and CPSF6 (Yue et al., 2018). Consistent with an earlier report, knockdown of METTL3 or VIRMA was found to encourage the usage of distal APA sites, thus lengthening the 3′ UTR of m^6^A-rich mRNAs. In contrast, CPSF5 knockdown elicits an opposite effect on the length of the 3′ UTR of m^6^A-marked mRNAs (Yue et al., 2018).

#### N^6^-Methyladenosine Promotes Nuclear Export

Considerable amount of nuclear export of mRNAs is regulated by the THO/TREX complex and the nuclear export factor heterodimer NXF1/P15 (Lesbirel and Wilson, 2019). Evidence is accumulating for the role of m^6^A modification in mRNA nuclear export. Knockdown of METTL3 resulted in delayed nuclear export of specific mRNAs of clock genes (Fustin et al., 2013), indicating the requirement of m^6^A methylation for specific mRNA nuclear export. Conversely, knockdown of ALKBH5 increased the cytoplasmic accumulation of polyA mRNAs (Zheng et al., 2013). Moreover, VIRMA was observed to interact with the mRNA export factor ALYREF, and its downregulation led to defective mRNA export (Masuda et al., 2005).

Interestingly, several TREX components associate with the components of the core m^6^A machinery (METTL3-METTL14-WTAP-VIRMA), and TREX also enhances the association of m^6^A reader YTHDC1 with the mRNA. Knockdown of YTHDC1 also resulted in reduced nuclear export of specific mRNAs (Lesbirel et al., 2018). Taken together, the abovementioned literature demonstrates that m^6^A modification factors promote mRNA nuclear transport through physical interaction with the mRNA transport machinery.

#### N^6^-Methyladenosine Enhances mRNA Degradation

Numerous recent studies suggest that impaired m^6^A writer complex function reduces m^6^A modification levels and raises mRNA stability, indicating that m^6^A methylation drives mRNA degradation (Batista et al., 2014; Schwartz et al., 2014; Wang X. et al., 2014; Wang Y. et al., 2014; Park et al., 2019). Mechanistically, m^6^A-containing mRNA recruits YTHDF2, which is followed by the translocation of the YTHDF2–mRNA complex from the translation machinery to P-bodies, leading to the degradation of YTHDF2-targeted mRNA. As a result, mRNA targets have increased half-life following YTHDF2 knockdown (Wang X. et al., 2014). It has been clearly demonstrated that YTHDF2 enhances m^6^A-modified mRNA decay through recruiting CCR4-NOT deadenylase complex *via* the N-terminus of YTHDF2 and reveals an underlying mechanism by which YTHDF2 regulates degradation of m^6^A-modified mRNAs (Du et al., 2016).

In a recent study, it was reported that some m^6^A-modified mRNAs interact with YTHDF2 to undergo decay in an RNase P/MRP-dependent manner and in which HRSP12 serves as a bridge between YTHDF2 and RNase P/MRP (Park et al., 2019). The interaction of human YTHDF2 and HRSP12 was first hinted by the association between their respective yeast homologs Pho92 and Mmf1 (Krogan et al., 2006). It was found in an immunoprecipitation experiment that HRSP12 links YTHDF2 and RNase P/MRP and that the N-terminus of YTHDF2 is required to interact with HRSP12 (Park et al., 2019). Of note, the subset of m^6^A-modified mRNAs, whose decay depends on YTHDF2–HRSP12–RNase P/MRP complex, contains a specific HRSP12-binding motif proximally upstream of the YTHDF2-binding motif, while the RNase P/MRP cleavage site is downstream and close to the YTHDF2-binding motif (Park et al., 2019). Therefore, this study discloses at least two mechanisms involved in the degradation of YTHDF2-associated m^6^A RNAs: HRSP12-RNase P/MRP-dependent and CCR4-NOT complex-dependent.

#### N^6^-Methyladenosine Modulates Translation

The m^6^A reader YTHDF1 enhances translation efficiency *via* interaction with eIF3A/eIF3B, and the YTHDF1-regulated translation likely hinges on eIF4G-dependent loop formation (Wang et al., 2015). According to Meyer et al. (2015), 5′ UTR m^6^A elevates cap-independent translation through recruiting the 43S complex to form 48S initiation complex in the absence of the cap-associating complex, eIF4F. This mechanism is important for cells to bypass 5′ cap-binding factors to enhance translation under stress conditions (Meyer et al., 2015). Moreover, heat stress-induced cytoplasmic-to-nuclear translocation of YTHDF2 is required for maintaining 5′ UTR m^6^A levels *via* competing for binding of the demethylase FTO to m^6^A sites, which further promotes cap-independent translation (Zhou et al., 2015). YTHDF1 preferentially binds to m^6^A marks in 3′ UTR of the oncogene *CDCP1* mRNA and promotes translation by increasing the amount of polysome-bound (translationally active) *CDCP1* transcripts (Yang et al., 2019).

Of note, METTL3 is also involved in m^6^A-enhanced mRNA translation through its role as an m^6^A reader in several ways. It promotes mRNA translation *via* physical association with the translation initiation complex (Lin et al., 2016). It was found that promoter-associated METTL3 regulates m^6^A methylation inside the coding region and improves mRNA translation through relief of ribosome stalling (Barbieri et al., 2017).

Besides promoting translation efficiency, m^6^A modification also plays an important role in regulating alternative translation (Zhou J. et al., 2018). It has been reported that widespread alternative translation occurs under various nutrient conditions, but the underlying mechanism is unclear (Gao et al., 2015). Recently, Zhou J. et al. (2018) found that m^6^A modification in the 5′ UTR modulates the selection of start codon globally, hence driving alternative translation. As representative examples, *Atf4* depends on decreased m^6^A modification of the upstream open reading frame 2 (uORF2) to improve the translation of the major isoform, and *Gadd45g* heightens the translation of the major isoform by lowering the m^6^A modification of the 5′ UTR (Zhou J. et al., 2018).

#### N^6^-Methyladenosine Methylation Increases the Phase Separation Capacity of mRNA

Only until recently has it become clearer how m^6^A modification drives mRNA fate and why the consequence of m^6^A modifications can vary in various scenarios. According to Ries et al. (2019), the m^6^A readers, YTHDF1−3, experience liquid–liquid phase separation (LLPS). The mRNAs with multiple m^6^A marks serve as a scaffold to bind with YTHDF readers *via* their low-complexity regions (LCRs). The mRNA–YTHDF complexes are then transported into various phase separators, like P-bodies, stress granule, and neuronal granules. The study suggests that the number and allocation of m^6^A modifications in mRNAs remodel the transcriptome of different phase-separated compartments. The efficacy of m^6^A modification-dependent modulation of an mRNA is likely governed by signals regulating the ability of YTHDF protein involved in LLPS formation (Ries et al., 2019).

## N^6^-Methyladenosine Modification Prominently Regulates Brain Development and Function

**FIGURE 3:**
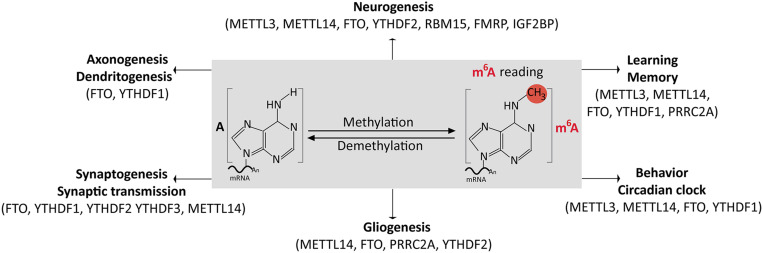
An illustration summarizing the role of N^6^-methyladenosine (m^6^A) in brain development and function. The functions of the various m^6^A-related factors involved in the proliferation and differentiation of neural precursors, neuronal maturation, production of glia, synapse formation during brain development, and common brain physiology are shown.

Evidence for the role of m^6^A signaling in modulating the development of the brain and its functions has accumulated in recent years, and the quest for extending the frontier is of great interest. Several investigations have revealed that the various factors that come together to form the m^6^A methylation machinery exert notable effect(s) on specific aspects of brain morphogenesis to permit optimal neural function, as summarized in Table 1. Conversely, the dysregulation of the m^6^A methylation machinery is known to elicit perturbations in the neural transcriptome, which have implications for defective development and dysfunction of the brain. The integrity of the m^6^A machinery functionality is of high priority in cells to the extent that simply ablating its cofactors can have significant consequences for brain development disturbance, as exemplified by the importance of Exosc10-mediated regulation of mRNA stability in forebrain development (Ulmke et al., 2021). The sections below discuss how specific factors associated with the m^6^A methylation machinery drive neural development, functional adaptation, and plasticity of the brain (Figure 3).

**TABLE 1:** m^6^A mRNA methylation factors and their role in brain development and function.

Effector	Experimental manipulation	Phenotype	Mechanisms	References
**Neurogenesis**				
METTL3	*Mettl3^fl/fl^; Nestin-Cre*	Prolongation of the cell cycle of RGCs and protraction of embryonic cortical neurogenesis	m^6^A depletion caused increased stability of NSC transcripts	Yoon et al. (2017)
METTL14	*Mettl14^fl/fl^; Nestin-Cre*	Reduced NSC proliferation and precocious NSC differentiation; loss of late-born neurons during cortical neurogenesis	Stabilization of CBP and p300 transcripts; H3K27me3-mediated transcription suppression of NSC proliferation genes; upregulation of H3K27ac in differentiation-related genes	Wang Y. et al. (2018)
RBM15	OE of *RBM15*	NSC delamination	Suppression of BAF155-dependent gene expression	Xie et al. (2019)
FTO	KO of *FTO*	Decrease in adult NSC proliferation and defective hippocampal neurogenesis	Impairment of BDNF and MAPK signaling	Li L. et al. (2017); Spychala and Ruther (2019)
YTHDF2	*Ythdf2 ^fl/fl^; Cre (ubiquitously)*	Decreased proliferation and differentiation capabilities of NSCs; less Tbr2+ bIPs; Reduced CP thickness	Promotion of m^6^A-dependent degradation of neurodevelopment-related transcripts	Li M. et al. (2018)
FMRP	KO of *Fmr1*	Nuclear retention of neurogenic mRNAs; prolonged cell cycle progression in the postnatal mouse brain	Unknown	Edens et al. (2019)
Exosc10	*Exosc10^fl/fl^; Foxg1-Cre; Emx1-Cre*	Apoptosis-mediated cortical agenesis	Mediates degradation of *Bbc3* and *Aen* mRNAs, which are effectors of apoptosis	Ulmke et al. (2021)
**Gliogenesis**				
METTL14	*Mettl14^fl/fl^; Olig2-Cre*	Decrease in oligodendrocytes maturation; cortical hypomyelination	Alters alternative splicing and expression of *Nfasc155*	Xu et al. (2020)
METTL14	*Mettl14; Nestin-Cre*	Reduced number of s100β+ astrocytes	Unknown	Yoon et al. (2017)
FTO	*FTO^fl/fl^; Olig2-Cre; Nestin-Cre*	Loss of OPCs and Sox10+ cells; cortical hypomyelination	Promotes *Olig2* mRNA degradation	Wu et al. (2019)
PRRC2A	*Prrc2a; Nestin-Cre; Olig2-Cre*	Loss of OPCs and mature oligodendrocytes; cortical hypomyelination	Gene targeting of *Prrc2a* by *olig2* mRNA	Wu et al. (2019)
PRRC2A	*Prrc2a; Nestin-Cre;*	Reduced proliferation capacity and number of astrocytes	Competitive expression with YTHDF2	Wu et al. (2019)
**Axonogenesis, dendritogenesis, synaptic plasticity**				
YTHDF1 YTHDF3	KD of *Ythdf1* and *Ythdf3*	Abnormal dendritic spine morphology	Inhibition of *Apc* mRNA translation	Merkurjev et al. (2018)
METTL14	*Mettl14^fl/fl^; D1R-Cre*	Abnormal excitability of striatal neurons	Unknown	Koranda et al. (2018)
FTO	KO of *FTO*	Defective synaptic plasticity	Unknown	Hess et al. (2013)
YTHDF1 YTHDF3	KD of *Ythdf1* and *Ythdf3;* CRISPR/Cas9-based KO of *Ythdf1*	Suppression of neuronal excitability	Not clear	Merkurjev et al. (2018); Shi et al. (2018)
**Learning and behavior**				
METTL3	OE of *METTL3*	Improved long-term memory consolidation	Unknown	Zhang Z. Y. et al. (2018)
METTL14	*Mettl14^fl/fl^; D1R-Cre*	Impaired striatum-mediated behavior patterns	Unknown	Koranda et al. (2018)
FTO	CRISPR/Cas9 or shRNA-mediated KD of *FTO*	Learning disabilities; defective memory processing and verbal fluency	Not clear	Ho et al. (2010); Benedict et al. (2011); Widagdo et al. (2016); Li L. et al. (2017); Walters et al. (2017); Sun et al. (2019)
PRRC2A	*Prrc2a; Nestin-Cre; Olig2-Cre*	Cognitive defects due to cortical hypomyelination	Unknown	Wu et al. (2019)
YTHDF1	CRISPR/Cas9-based KO of *Ythdf1*; KD of *Ythdf1*	Defective long-term potentiation and synaptic transmission in hippocampus; behavioral defects	Unknown	Shi et al. (2018)
**Circadian clock**				
METTL3	KD of *Mettl3*	Elongation of circadian period	Defective processing of *Per2* and *Arntl* (clock genes) mRNAs	Fustin et al. (2013)
**Stress response**				
FTO	*FTO^fl/fl^; Camk2a-Cre; Nex-CreERT2*	Reduced ability to cope with stress	Unknown	Engel et al. (2018)
METTL3	*Mettl14^fl/fl^; Camk2a-Cre; Nex-CreERT2*	Reduced ability to cope with stress	Unknown	Engel et al. (2018)

*KO, knockout; KD, knockdown; OE, overexpression; fl/fl, double floxed; RGCs, radial glial cells; OPCs, oligodendrocyte precursor cells; NSCs, neural stem cells; CRISPR, clustered regularly interspaced short palindromic repeats; m^6^A, N^6^-methyladenosine.*

### N^6^-Methyladenosine Modification Is Indispensable for Neurogenesis in the Brain

Neurons are produced through the process of neurogenesis, which entails specification and proliferation of NSCs, and the differentiation of such neural progenitors into neuroblasts, which undergo maturation to become functional neurons. It has been shown that the dynamic addition of m^6^A to gene transcripts in the multipotent NSCs greatly influences cortical neuroprogenitor competence and the generation of neurons during brain development (Yao et al., 2016; Boles and Temple, 2017; Yoon et al., 2018; Zhou H. et al., 2018; Rockwell and Hongay, 2019). Dysregulation of writers, erasers, and readers of m^6^A has been reported to cause notable perturbations in the cell cycle progression, proliferation, and differentiation of NSCs in the developing and adult brain.

#### Effect of N^6^-Methyladenosine Writers on Neurogenesis in the Brain

So far, it has been shown that ablation of the m^6^A writer METTL3 or its cofactor METTL14 in cortical neuroepithelium or isolated cortical NSC results in prolonged cell cycle dynamics of cortical neuroprogenitors and their precocious differentiation into neuronal or neurogenic cells (Batista et al., 2014; Yoon et al., 2017; Wang Y. et al., 2018). Detailed analysis through m^6^A sequencing revealed that gene transcripts involved in the cell cycle of neural cells, production of neurons, and neuronal differentiation are enriched with m^6^A tags. Interestingly, the decay of such mRNAs is promoted in the absence of METTL3 and METTL14 (Yoon et al., 2017), meaning that METTL3 and METTL14 are key players in driving neurogenesis *via* the stabilization of gene transcripts critical for neurogenesis in the brain. For example, loss of m^6^A due to deletion of METTL3 in mouse cerebellum resulted in overt hypoplasia partly attributable to apoptosis of cerebellar granule cells (Wang C. X. et al., 2018). Key downstream effects of m^6^A on genes important for neurogenesis include the modulation of histone modification in the promoter environment of NSC proliferation- and differentiation-related gene loci (Wang Y. et al., 2018). In the absence of METTL14, the transcription-suppressing histone mark H3K27me3 is upregulated on genes involved in cell proliferation, whereas differentiation-related genes show an increase in the transcription activation histone mark H3K27ac when METTL14 is deficient (Wang Y. et al., 2018). Lack of METTL3 in the developing brain can also cause the aforementioned histone alterations, at least in terms of H3K27me3 enhancement, which can cause transcription repression (Chen J. et al., 2019). This is possible because in the absence of METTL3, which leads to a reduction in m^6^A levels, the polycomb repressor complex becomes hyperactive due to derepression of its core methlytransferase factor Ezh2 (Chen J. et al., 2019).

RBM15, a core component of the m^6^A writer complex (Figure 1A), is a potential regulator of cortical neurogenesis due to its distinctive expression in the cortical germinative zone and cortical plate of the developing mouse cortex (Xie et al., 2019). Knockdown of RBM15 in neurons *in vitro* promoted endogenous expression of the chromatin remodeling factor BAF155 (Xie et al., 2019), which is a known key regulator of cortical development (Nguyen et al., 2016, 2018; Narayanan et al., 2018). This profound effect can be linked to a significant reduction in cellular levels of m^6^A due to the inactivation of RBM15 (Knuckles et al., 2018). However, overexpression of RBM15 *in vivo* was found to promote delamination of radial glial cells in the cortical ventricular zone by suppressing the expression BAF155 and, hence, BAF155-dependent gene expression program supportive for cortical development (Xie et al., 2019). The role of RBM15 in cortical neurogenesis further highlights the contribution of m^6^A methyltransferase in brain development.

#### N^6^-Methyladenosine Erasers Regulate Neurogenesis in the Brain

Erasers of the m^6^A mark (FTO and ALKBH5) can also exert a regulatory effect on the process of neurogenesis given their prominent expression in neurons (Li L. et al., 2017; Yoon et al., 2017; Spychala and Ruther, 2019; Du et al., 2020). Whereas FTO displays the highest expression level late in brain neurogenesis (Li L. et al., 2017; Yoon et al., 2017), ALKBH5 expression decreases in the course of brain development (Du et al., 2020). This may have implications for their roles in the spatiotemporal regulation of neurogenesis during brain development. Indeed, it was reported that FTO deficiency in the adult mouse brain induces signal transducer and activator of transcription (STAT)3 pathway activation *via* its modulators platelet-derived growth factor receptor (PDGFR)α and suppressor of cytokine signaling (SOCS)5 in an m^6^A-dependent manner (Cao et al., 2020). As a result, a transient increase in the proliferation and differentiation of adult NSCs was observed in the FTO mutant brain, with implications for adult neurogenesis inhibition in the long term (Cao et al., 2020). It was also observed that FTO deletion in adult mouse brain impairs brain-derived neurotrophic factor (BDNF) and mitogen-activated protein kinase (MAPK) signaling pathways, leading to a reduction in adult NSC proliferation and neurogenesis in the hippocampal formation (Li L. et al., 2017; Spychala and Ruther, 2019). Although these studies report diverging effects of FTO loss on adult NSC proliferation, they both show a resultant effect of adult neurogenesis reduction. We think that, while being mindful of the low level of FTO expression in the early developing cortex, conducting an investigation on how FTO regulates corticogenesis in the course of development may lend clarity to how it mechanistically impacts cortical neurogenesis.

#### Notable N^6^-Methyladenosine Readers in Cortical Neurogenesis

Protein factors that act as readers of the m^6^A mark have also been shown to have a profound effect on neurogenesis in the brain. For instance, the m^6^A reader YTHDF2 has been reported to be indispensable for corticogenesis in mouse. Conditional knockout of YTHDF2 in the mouse neocortical neuroepithelium resulted in a reduction in the proliferation and differentiation of the *Ythdf2*^–^*^/^*^–^ neuroprogenitor cells (Li M. et al., 2018). This phenotype may have mechanistic underpinnings, including abnormal upregulation of genes that inhibit the JAK–STAT signaling pathway, due to increased stability of such gene transcripts in the absence of YTHDF2 (Li M. et al., 2018). Yet, it seems that the induction of neural fate in pluripotent stem cells requires downregulation of YTHDF2, leading to the stability and expression of neural gene transcripts (Heck et al., 2020). We are of the opinion that the functional consequence of the m^6^A reading by YTHDF2 may be contextually variable along the cortical development axis such that reduced dosage may support neural cell fate specification, whereas its increased activity/expression is necessary for later cortical neurodevelopment.

Another m^6^A reader, FMRP, was identified to be critical for neural progenitor cell proliferation. Mice lacking *Fmr1* displayed prolonged cell cycle progression. As a result, proliferation of neural progenitors extended into postnatal stages of brain development (Edens et al., 2019). Of note, it was observed that nuclear export of m^6^A-modified neurogenic mRNAs readable by FMRP is defective, leading to retention of such neurodifferentiation gene transcripts in the nucleus of the *Fmr1*-deficient neural progenitor cells (Edens et al., 2019). Lastly, the m^6^A reader protein Imp (IGF2BP) was identified as a key regulator of NSC proliferation rate through the stabilization of *Myc* mRNA in *Drosophila* brain neuroblasts (Samuels et al., 2020).

Together, the m^6^A machinery has been identified to play critical roles in brain morphogenesis by regulating the proliferation of neural progenitor cells and the production of neurons. As such, hypomethylation due to METTL3 or METTL14 deficiency and aberrant m^6^A reading or erasure in the embryonic or adult brain can precipitate phenotypes, including defective transcriptional prepatterning, abnormal neuroprogenitor pool, impaired neurogenesis, and cortical hypoplasia (Yoon et al., 2017), which can engender deficits in brain structure and function.

### N^6^-Methyladenosine Signaling Is Essential for Gliogenesis in the Brain

The process of generating glial cells constitutes gliogenesis. Brain neuroglia include astrocytes and oligodendrocytes, which are derived from the neuroepithelium. During cortical development, a switch from neurogenesis to gliogenesis coincides with a decrease in m^6^A modification of proneural genes (Donega et al., 2018). Although m^6^A enrichment in glial cells is less than that observed in neurons (Chang et al., 2017), a few studies have uncovered the importance of the m^6^A methylome in brain gliogenesis, at least for astrocyte production (astrogenesis) and oligodendrocyte generation (oligodendrogenesis).

#### Regulation of Glia Production in the Brain by an N^6^-Methyladenosine Writer-Related Factor

It was observed that loss of METTL14-mediated m^6^A writing in the mouse cortex leads to hypomyelination that can be linked to a reduction in the number of (mature) oligodendrocytes (Xu et al., 2020). The loss of oligodendrocytes caused by the absence of METTL14 in the brain likely did not emanate from abnormal specification or proliferation of oligodendrocyte precursor cells (OPCs) (Xu et al., 2020). Notably, the transcriptome of OPCs and oligodendrocytes is altered following METTL14 deletion, with possible impact on gene expression programs critical for oligodendrocyte lineage progression (Xu et al., 2020). Lack of METTL14 has also been reported to disrupt astrogenesis. Indeed, s100β-expressing astrocytic progenitors were found to be reduced in the METTL14 knockout mouse cortex at postnatal stage 5 (Yoon et al., 2017). It would be interesting to investigate whether other m^6^A writer-related factors, including METTL3, have roles to play in cortical gliogenesis.

#### The N^6^-Methyladenosine Eraser FTO Regulates Glia Production in the Brain

m^6^A-mediated RNA methylation dynamics under the guild of FTO is known to influence oligodendrogenesis *via* modulation of the half-life of *Olig2* mRNA (Wu et al., 2019). Olig2 is a central factor indispensable for oligodendrocyte lineage progression (Liu et al., 2007). Specifically, FTO was reported to regulate the degradation of *Olig2* transcripts *via* removal of m^6^A tags installed on the *Olig2* mRNA. The stability of *Olig2* transcripts in OPCs deficient in FTO was thus seen to increase. In effect, the white matter in FTO mutant mouse brain was characterized by hypomyelination (Wu et al., 2019).

#### Involvement of N^6^-Methyladenosine Readers in Glia Production in the Brain

The m^6^A reader PRRC2A is known to be essential for oligodendrogenesis. It prominently regulates the specification, proliferation, and differentiation of oligodendroglia and the ability of oligodendrocytes to carry out myelination in the brain (Wu et al., 2019). More specifically, abolishing PRRC2A function in cortical NSCs or precisely in oligodendroglial lineage caused significant loss of OPCs (PDGFRα+ cells), Sox10+ cells, and mature oligodendrocytes (CC1+Oilg2+), which culminated in hypomyelination in the PRRC2A mutant brain (Wu et al., 2019). Interestingly, deletion of PRRC2A also affects astrogenesis, although slightly. Deficiency of PRRC2A in mouse brain caused a reduction in the proliferative capacity of astrocytes, leading to a reduced number of astrocytes in the mutant mouse brain (Wu et al., 2019). The additional role of PRRC2A in regulating the production of astrocytes in the brain during development may hinge on its interaction with YTHDF2, another m^6^A-binding protein, such that lack of either m^6^A reader augments the expression of the other to influence gliogenesis (Wu et al., 2019).

The competitive relationship between PRRC2A and YTHDF2 makes it complex to explain or reconcile the observation that glial fibrillary acidic protein (GFAP) expression, which can indicate astrocytic cells, was found to be dramatically reduced in neurospheres derived from the E14.5 *Ythdf2^–/–^* forebrain NSC. Such GFAP+ *Ythdf2^–/–^* cells also displayed abnormally branched processes (Li M. et al., 2018). Thus, further investigation is required to elucidate the role of YTHDF2 in brain gliogenesis and how the function of PRRC2A features in the regulatory pathway.

### N^6^-Methyladenosine Effectors Regulate the Formation of Neural Processes and Synapses

The developing and adult brain is characterized by the outgrowth of dendrites and axons of neurons known to form neural connections called synapses. Interestingly, synapses are enriched with m^6^A, which modulates dendrite formation (dendritogenesis), axonogenesis, and synaptic growth (synaptogenesis) and activity (reviewed in Li et al., 2019; Dermentzaki and Lotti, 2020). m^6^A-based transcriptome profiling of the mouse brain (cortex and cerebellum) showed enrichment of m^6^A modification linked to dendrite and dendritic spine, axon and axon guidance, and synaptogenesis and synaptic transmission (Chang et al., 2017).

Distinctive localization of the YTHDFs, FTO, and METTL14 in dendrites of hippocampal neurons in culture and cortical neurons suggests the involvement of these m^6^A-regulatory factors in the development of neural dendrites. Indeed, *Ythdf1* and *Ythdf3* knockdown in such cultured neurons resulted in abnormal dendritic spine (Merkurjev et al., 2018). Axons are also enriched with FTO, which can be translated locally. As such, FTO ablation in axons resulted in upregulation of m^6^A levels, leading to a reduction in *Gap-43* mRNA translation in axons of cultured dorsal root ganglion neurons (Yu et al., 2018). Yet, GAP-43 is a key factor involved in axon growth in neural tissues (Skene et al., 1986). In effect, the neurons lacking FTO displayed axon elongation repression (Yu et al., 2018). The m^6^A reader YTHDF1 was also reported to influence axon formation by binding and promoting the translation of the axon guidance receptor Robo3.1, which directs spinal commissural axons in crossing the midline, in an m^6^A modification-dependent manner (Zhuang et al., 2019). Together, these observations may have implications for perturbed axonogenesis in the brain lacking optimal m^6^A modification due to ablation of FTO or YTHDF1. At least in the case of the m^6^A-regulatory protein PRRC2A, it was found that axons that form the corpus callosum, a brain midline structure, are hypomyelinated and appeared hypoplastic in the PRRC2A-deleted mouse brain (Wu et al., 2019).

Given the enrichment of m^6^A marks and related proteins in neural processes, it is not surprising that synapses are endowed with m^6^A-modified mRNAs, especially postsynaptic transcripts in the mouse brain (Chang et al., 2017). The high localization of m^6^A-modified mRNAs in synapses reflects the possible impact of the m^6^A epitranscriptome on the structure, maturation, and function of synapses (Chang et al., 2017; Merkurjev et al., 2018; Yu et al., 2018; Zhuang et al., 2019). As a result, selective ablation of YTHDF1 and YTHDF3 in the cultured hippocampal neurons caused excitatory synaptic transmission suppression (Merkurjev et al., 2018; Shi et al., 2018). In addition, synapses formed by neurons lacking YTHDF2 appeared abnormal (Li M. et al., 2018), and synaptic transmission-related transcripts are hypermethylated in dopaminergic neurons with defective synaptic plasticity implication in the FTO-deficient mouse midbrain (Hess et al., 2013). Another indication of synapse malformation and synaptic plasticity impairment due to m^6^A dysregulation was observed in METTL14-deleted striatal neurons, in which METTL14 abrogation resulted in aberrant neuronal excitability (Koranda et al., 2018). Given that Nito, the *Drosophila* version of RBM15, also regulates synaptic growth through regulation of axonogenesis (Gu et al., 2017), it would be interesting to investigate whether indeed RBM15 is involved in synaptogenesis in the mammalian brain.

### Cognition and Behavior Are Modulated by N^6^-Methyladenosine Signaling

The brain’s ability to process and store information and form or control behavior patterns has been shown to be greatly regulated by posttranscriptional modification of mRNA involved in brain development (reviewed in Jung and Goldman, 2018; Leighton et al., 2018; Noack and Calegari, 2018). Prominently emerging among these new (epitranscriptomic) levels of brain function regulation is m^6^A modification of mRNA in the brain. Various studies in mouse models have revealed the involvement of the m^6^A machinery-related factors in cognition and behavior (reviewed in Nainar et al., 2016; Chokkalla et al., 2020). The role of m^6^A in the regulation of learning and behavior may be partly explained by the previously discussed role of m^6^A in synaptogenesis and synaptic transmission (Weng et al., 2018).

#### N^6^-Methyladenosine Writers Involved in Memory and Behavior

In a recent study by Zhang F. et al. (2018), it was found that the enrichment of METTL3 in the mouse hippocampus is supportive for memory consolidation *via* the promotion of neuronal early-response gene translation. Therefore, mice lacking METTL3 in the hippocampus displayed impaired long-term potentiation with attendant reduced ability to consolidate memory. Interestingly, long-term memory consolidation is demonstrably augmented following METTL3 overexpression in the dorsal hippocampus of the wild-type mouse brain (Zhang Z. Y. et al., 2018). The m^6^A writer function of METTL14 is reported to be important for learning and behavior mediated by the striatum. Without affecting the number or morphology of striatal neurons, loss of METTL14 in striatopallidal and striatonigral neurons caused alterations in the transcriptome, eliciting increased neuronal excitability and spike frequency adaptation reduction, which possibly culminated in impairment of striatum-dependent behavior patterns (Koranda et al., 2018).

#### The N^6^-Methyladenosine Eraser FTO Regulates Learning and Behavior

Accumulation of m^6^A in the brain can affect its learning capacity and behavior. By regulating adult neurogenesis in the mouse hippocampus, FTO has been identified to play a pivotal role in learning (Li L. et al., 2017). Hypermethylation in the mouse brain or hippocampus caused by FTO functional loss was observed to call forth learning disabilities in mice, including increased fear memory consolidation (Widagdo et al., 2016; Walters et al., 2017). Additional evidence indicating the role of FTO in learning and behavior includes a study in which mice deficient in FTO were reported to exhibit behaviors consistent with depression and anxiety (Sun et al., 2019). Moreover, available data show that memory processing and verbal fluency may be affected in individuals with FTO ablation in the brain (Ho et al., 2010; Benedict et al., 2011).

#### Readers of N^6^-Methyladenosine Modulate Learning and Memory

Cognitive deficits have been implicitly linked to lack of function of the m^6^A reader PRRC2A, whose absence caused hypomyelination, leading to the cognitive anomalies in the mouse brain (Wu et al., 2019). Evidence indicating a more direct importance of an m^6^A reader in learning and memory was obtained when YTHDF1 was deleted in the adult mouse brain. It was found that neuronal stimuli can evoke translation of gene transcripts readable by YTHDF1 to facilitate learning and memory (Shi et al., 2018). Hence, silencing of YTHDF1 in the mouse hippocampus resulted in defective long-term potentiation and impaired synaptic transmission in the hippocampus, which did not allow normal learning and memory processing, and the defects were rescuable by YTHDF1 re-expression in the YTHDF1 mutant brain (Shi et al., 2018).

### Stress Response Is Regulated by Factors of the N^6^-Methyladenosine Machinery

The brain plays a central role in stress response. In responding to stress, a host of gene expression programs is activated in the brain, leading to the secretion of several neuropeptides (de Kloet et al., 2005). Vulnerability to stressful stimuli and the response mechanism can have implications for neuropsychiatric anomalies under abnormal regulatory conditions. Thus, the transcriptomic stress response system is particularly crucial in maintaining homeostasis following exposure to stress.

Epigenetic mechanisms are known to play central roles in stress response (McEwen et al., 2015), and the epitranscriptome is an emerging gene expression regulation domain for stress modulation (Harvey et al., 2017). A putative role for m^6^A in the regulation of stress response is evidenced by the presence of glucocorticoid response elements upstream the transcription start site of genes that encode for enzymes involved in m^6^A modification (Engel et al., 2018). Additionally, nuclear localization of YTHDF2 precipitated by heat stress results in dynamic methylation of the 5′ UTR of newly synthesized mRNAs (Zhou et al., 2015). By limiting FTO, YTHDF2 is able to preserve methylation in the 5′ UTR of heat stress-induced mRNAs (Zhou et al., 2015).

In chick, upregulation of FTO in the brain (hypothalamus) may be a mechanism to afford thermoregulation in heat stress conditions (Kisliouk et al., 2020). However, following acute restraint stress, the mouse prefrontal cortex and amygdala displayed m^6^A hypomethylation and hypermethylation, respectively (Engel et al., 2018). Fear-induced stress can cause downregulation of FTO, leading to elevation of m^6^A in the prefrontal cortex and hippocampus of the mouse (Walters et al., 2017). Mice lacking METTL3 or FTO are unable to cope with stress (Engel et al., 2018). A general effect that may be caused by stress-induced alteration in m^6^A modification is the suppression of mRNAs involved in synaptic plasticity and brain morphogenesis (Engel et al., 2018). Together, the above observations indicate a putative role for m^6^A modulation in the human brain during stressful insults.

## Neurological Disorders Attributable to Defective N^6^-Methyladenosine Modification in the Brain

Emerging evidence shows that a number of syndromic and non-syndromic neurological disturbances can be linked to m^6^A methylome dysregulation in the brain (Engel and Chen, 2018). This is not surprising, given the previously discussed extensive role of m^6^A in brain neurodevelopment (Figure 3). The m^6^A ubiquity in the brain implies that neural perturbations due to m^6^A dysregulation are likely to be complex and multifactorial in terms of downstream causatives. Neurologic problems so far identified to be caused by genetic variants of m^6^A modification factors can be broadly characterized as neurodevelopmental, neurodegenerative, or neuropsychiatric. Specifically, these include Parkinson’s disease (PD), Alzheimer’s disease (AD), autism, Smith–Magenis syndrome, schizophrenia, and depression (Table 2). The following subsections discuss the role of m^6^A and associated factors in neurological disorders of the brain.

**TABLE 2:** Brain disorders associated with m^6^A dysregulation.

Neurological disorders	Experimental system	m^6^A factor(s) implicated	References
**Neurodevelopmental disorders**			
Microcephaly	GWAS; KO mice	*Fto* deletion; *METTL5* frameshift	Richard et al. (2019)
Fragile X Syndrome	GWAS	SNP in *FMRP*	Verkerk et al. (1991); Dictenberg et al. (2008)
Cerebellar ataxia	KO mice; KO *Drosophila*	Deletion of *Ythdc1*, *Mettl3*, *Alkbh5*	Fernandez-Funez et al. (2000); Ma et al. (2018); Wang C. X. et al. (2018)
Smith–Magenis syndrome	Genetic analysis in mouse	*Alkbh5* deletion	Ricard et al. (2010)
Intellectual disability	GWAS	*METTL5* frameshift	Richard et al. (2019)
Autism spectrum disorder	GWAS	Mutations in *FMR1*	Reddy (2005); Edupuganti et al. (2017); Kaufmann et al. (2017)
**Neurodegenerative disorders**			
Parkinson’s disease	6-OHDA treatment of PC12 cells and rats; KO mice	*Fto* deletion or inhibition	Hess et al. (2013); Chen X. C. et al. (2019)
Alzheimer’s disease	GWAS	SNP in *FTO*	Ho et al. (2010); Keller et al. (2011); Reitz et al. (2012)
Amyotrophic lateral sclerosis	GWAS	SNP in *FTO;* SNP in *HNRNP (A2B1 and A1);* SNP in *RBM15*	Kim et al. (2013); Cooper-Knock et al. (2017); Mitropoulos et al. (2017)
Cerebellar ataxia	KO mice; KO *Drosophila*	Deletion of *Ythdc1*, *Mettl3*, *Alkbh5*	Fernandez-Funez et al. (2000); Ma et al. (2018); Wang C. X. et al. (2018)
Multiple sclerosis	GWAS	SNP in *METTL1*	Mo et al. (2019)
**Neuropsychiatric disorders**			
Major depressive disorder	GWAS	SNP in *ALKBH5*; SNP in *FTO*	Samaan et al. (2013); Milaneschi et al. (2014); Du et al. (2015)
Schizophrenia	GWAS	SNP in *ZC3H13*	Oldmeadow et al. (2014)
Attention-deficit/hyperactivity disorder	GWAS	SNP in *FTO*	Velders et al. (2012); Choudhry et al. (2013)

*GWAS, genome-wide association studies; 6-OHDA, 6-hydroxydopamine; SNP, single-nucleotide polymorphism; KO, knockout; m^6^A, N^6^-methyladenosine.*

### Fragile X Syndrome

It has been identified that Fragile X syndrome (FXS) is the most common cause of inherited intellectual disorders and usually co-occurs with autism spectrum disorder (ASD). Patients present with features such as poor language development, abnormal behavior, and seizures, which are mainly clinical manifestations of neuronal excitation–inhibition imbalance (Hagerman et al., 2017; Kaufmann et al., 2017). Silencing of the *FMR1* gene, which leads to lack of FMRP expression, is the cause of FXS (Brown et al., 2001). The role of FMRP in multiple gene expression programs partly accounts for the syndromic nature of FXS (Hagerman et al., 2017). Synaptic abnormalities or loss of neuroplasticity caused by FMRP loss-of-function and perhaps YTHFC2 deficiency is a critical underlying mechanism that contributes to the etiology of FXS and associated ASD (reviewed in Liu et al., 2016; Bagni and Zukin, 2019).

### Parkinson’s Disease

Parkinson’s disease is a complex progressive neurodegenerative disorder mainly associated with death of dopamine-producing neurons in the midbrain (substantia nigra pars compacta) and aggregation of Lewy bodies in various brain regions. The main symptoms of PD include tremor and bradykinesia. Until now, the cause of PD is unknown, as many genetic and environmental risks are involved, making definitive diagnosis and treatment challenging (Kalia and Lang, 2015; Hayes, 2019).

Interestingly, m^6^A methylation deregulation caused by FTO abrogation, in the midbrain or in dopaminergic neurons, has been implicated in PD pathogenesis *via* impairment of neuronal activity and behavior response dependent on dopamine receptor types 2 and 3 (Hess et al., 2013). mRNAs involved in dopaminergic signaling are hypermethylated in the FTO-deficient mouse midbrain and striatum, leading to their decreased translation (Hess et al., 2013). It was found in another study that m^6^A may play a role in loss of dopaminergic neurons, which characterizes PD (Chen X. C. et al., 2019). The study reported that PC12 cells treated with 6-hydroxydopamine (6-OHDA) and the striatum of rat brain with 6-OHDA-induced PD display m^6^A modification downregulation, which is capable of inducing N-methyl-D-aspartate (NMDA) receptor 1 expression, alongside elevated oxidative stress and influx of Ca^2+^, culminating in cell death of dopaminergic neurons. Notably, FTO inhibition, and perhaps inhibition of ALKBH5, can attenuate 6-OHDA-induced PC12 cells apoptosis (Chen X. C. et al., 2019).

### Alzheimer’s Disease

The commonest cause of dementia worldwide is AD. It is mainly characterized by progressive (age-dependent) neurodegeneration in brain regions (especially in the temporal and frontal lobes), with key clinical features, including memory loss, behavior abnormalities, and eventual cognitive decline (reviewed in Weller and Budson, 2018; Soria Lopez et al., 2019). Errors in RNA metabolism can have implications for AD. As discussed further, studies in human populations and in mouse models have shown that specific dysregulations in m^6^A mRNA methylation contribute to AD pathogenesis.

Typically, m^6^A levels in various brain regions increase with aging, and this disposition was shown to likely have relevance for AD development (Shafik et al., 2021). Interestingly, while METTL3 is downregulated in AD brain (hippocampus), it was observed to have accumulated in the postmortem AD brain at levels comparable to the insoluble Tau protein therein (Huang H. et al., 2020). Immunohistochemistry of the entorhinal cortex of patients with AD showed selective deficiency in the expression of another m^6^A factor hnRNP-A/B, which probably underscores the alteration in alternative splicing in the AD brain (Berson et al., 2012). Moreover, FTO mis-expression is implicated in the development of AD. Carriers of the *FTO* variant rs9939609 were reported to display systematic deficits in brain volume consistent with brain atrophy in the elderly (Ho et al., 2010). Indeed, a population-based study found an association between the *FTO* variant rs9939609 and increased risk of AD (Keller et al., 2011). Reitz et al. (2012) reported an increased risk caused by some polymorphisms (rs11075997, rs11075996, rs17219084) in the *FTO* gene in AD cases among some investigated Caribbean Hispanics and Caucasians (Reitz et al., 2012). Reduced verbal fluency in obese and overweight elderly men, with unaffected general cognitive function, was attributed to bearing of the *FTO* A allele. Thus, the (dys)functional effect of *FTO* A allele mainly manifests in the frontal lobe of the brain to constitute AD (Benedict et al., 2011). These observations indicate perturbation of m^6^A signaling as a notable underlying factor in the pathophysiology of AD in humans.

*In vitro* and *in vivo* experimentations using mouse models have yielded results that further support the involvement of m^6^A mRNA methylation in AD. In one study, it was observed that knockdown of hnRNP A/B impaired alternative splicing in cultured neurons, which resulted in loss of dendrites, and caused memory impairment in mice that can be ascribed to aberrance in the cortical connectome (Berson et al., 2012). The level of hnRNP A/B increases with cholinergic excitation, whereas loss of cholinergic signaling was found to induce AD-like reduction in hnRNP levels in the cortex (Berson et al., 2012).

The AD brain of the APP/PS1 transgenic mouse has elevated levels of m^6^A in the hippocampus and cortex, which may be due to the increased expression of METTL3 and concurrent downregulation of FTO expression (Han et al., 2020). However, the expression of FTO was identified to be increased in the brain of the triple transgenic AD mouse (Li H. et al., 2018). This gives an impression of the complex nature of the mechanism through which FTO or other m^6^A-associated factors may drive the development of AD. In the case of FTO, a proposed mechanism is that it may promote the phosphorylation of Tau protein by encouraging a methylation scheme leading to stabilization of tuberous sclerosis complex 1 (TSC1) mRNA, which activates the kinase activity of the mammalian target of rapamycin (mTOR) (Li H. et al., 2018).

Interestingly, cognition in a mouse model of AD was observed to improve when FTO was conditionally deleted in neurons in the mouse brain with AD (Li H. et al., 2018). This makes FTO a prospective therapeutic candidate worth further investigation for its potential in slowing down the progression of AD or in remedying related symptoms.

### Amyotrophic Lateral Sclerosis

Amyotrophic lateral sclerosis (ALS) is a debilitating neurodegenerative disorder hallmarked by loss of motor neurons leading to skeletal muscle dysfunction and other clinical features, including psychological disorders and respiratory distress (Rowland and Shneider, 2001). It is believed to be idiopathic, with a greater percentage (∼90%) of cases being sporadic, while 5%−10% of cases are familial or inheritable (Kiernan et al., 2011). Studies have revealed the prominent role played by pathogenic mutation of factors associated with the RNA methylation machinery (Kim et al., 2013; Cooper-Knock et al., 2017; Mitropoulos et al., 2017).

By means of whole-genome sequencing, it became evident that m^6^A may be involved in the pathogenesis of ALS through FTO function alteration (Mitropoulos et al., 2017). Variants of *FTO* gene were thus associated with sporadic cases of ALS, which appears to be a founder effect among Greeks (Mitropoulos et al., 2017). In another key study, mutation in the prion-like domain of the m^6^A reader HNRNP (A2B1 and A1) was implicated in the pathogenesis of a familial ALS case (Kim et al., 2013). The work of Cooper-Knock et al. (2017) supports the involvement of RNA-binding protein mutations in ALS. Deleterious variants of *RBM15* gene or its paralog *RMB15B* were found to contribute to the pathogenesis of ALS (Cooper-Knock et al., 2017).

### Major Depressive Disorder

Major depressive disorder (MDD) is a common neuropsychiatric condition that is considered a biobehavioral syndrome with clinical characteristics including depressed mood, cognitive dysfunction, neurovegetative disturbance, and diminished interests. Females are known to be more affected by MDD than males. Multiple factors are known to cause MDD. Notable underlying causatives include genetic and environmental factors leading to alteration in the volume of the hippocampus and aberrant brain circuitry (Fava and Kendler, 2000; Flint and Kendler, 2014; Otte et al., 2016).

The m^6^A RNA methylome plays a role in the development of MDD (Engel et al., 2018). Genetic variants of FTO have been implicated in MDD, although heterogeneity in the associated phenotype is noteworthy (Milaneschi et al., 2014). In particular, it was found in a genome-wide association study that the *FTO* rs9939609 A variant is associated with a reduced risk of MDD (Samaan et al., 2013). A single-nucleotide polymorphism (rs12936694) in *ALKBH5* was also found to likely be the cause of MDD among the Chinese Han population in an association study (Du et al., 2015). Interestingly, by blocking the translocation of ALKBH5 into the nucleus, it was possible to attenuate depression-like behavior in the mouse due to attendant hypermethylation and subsequent degradation of fatty acid amide hydrolase mRNA in astrocytes (Huang R. R. et al., 2020).

## Therapeutic Prospects of CRISPR–Cas13-Mediated RNA Methylation Regulation in N^6^-Methyladenosine-Related Neurological Disease Treatment

While it seems intuitive that a simple strategy of traditional knockdown or overexpression of dysfunctional m^6^A factors in the epitranscriptome can correct pathologic alterations in the RNA methylation program, heterogeneity of the m^6^A methylome and, in some cases, the possible functional duplication or duality of the m^6^A writers, erasers, and readers, possess a challenge for the applicability of such solutions. To circumvent the aforementioned constraints, a system or tool capable of targeting defective m^6^A sites with high specificity should be considered. Such targeted approach to reversing disease-causing m^6^A modification can have therapeutic application if perfected.

**FIGURE 4:**
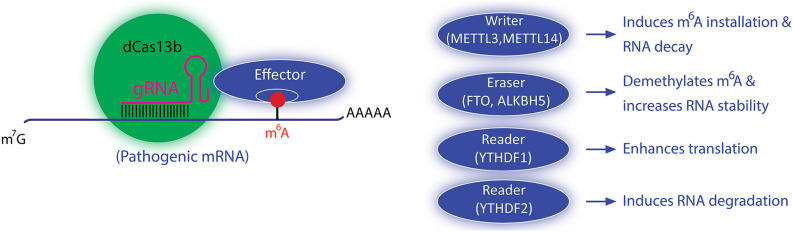
Schema showing application of clustered regularly interspaced short palindromic repeats (CRISPR)–dCas13 for N^6^-methyladenosine (m^6^A) editing. The m^6^A editing system is made by fusing deactivated Cas13b (dCas13b) with a guide RNA (gRNA) that can specifically target abnormally methylated messenger RNA (mRNA) (pathogenic mRNA) and coupling of CRISPR–dCas13 to an m^6^A factor to effect desired changes in m^6^A modification. Depending on the effector used, it is possible to induce m^6^A deposition, removal, or recognition (binding/reading), leading to the induction of degradation, translation enhancement, or increased stability of the target mRNA.

The discovery of the Cas13 family of proteins, which are able to target endogenous RNA, has opened up avenues to deliver specific effectors at single sites on gene transcripts (Abudayyeh et al., 2016). By associating clustered regularly interspaced short palindromic repeats (CRISPR) with a catalytically inactive form of Cas13 protein (dCas13), but having preserved RNA binding ability, (m)RNA can be targeted at specific nucleic acid loci with such designed programmable CRISPR–dCas13 system (Figure 4; Wang et al., 2019; Burmistrz et al., 2020). Here, we discuss various salient *in vitro* applications of the CRISPR–dCas13 system to achieve m^6^A editing (Table 3).

**TABLE 3:** Applications based on CRISPR–dCas13b system for targeted manipulation of m^6^A modification and fate of m^6^A-tagged mRNA.

System	Effect	Targeted mRNA	Reference
**Targeting m^6^A installation**			
dCas13b-METTL3 dCas13b-METTL14	Induces effective m^6^A incorporation in endogenous transcripts with increased specificity; regulates m^6^A-dependent mechanism for controlling transcript abundance; induces alternative splicing	*Actb, Gapdh, Foxm1, Sox2, Brd8, Znf638*,	Wilson et al. (2020)
dCas13b-CIBN CRY2PHR-METTL3-METTL14	Effect photoactivatable RNA m^6^A level upregulation	*TPT1*, *ACTB, TUG1*	Zhao et al. (2020)
**Targeting m^6^A removal**			
dCas13b-FTO	Demethylates m^6^A of targeted mRNAs to enhance their stability	*MALAT1, ASB2, HBV, HXB2*	Mo et al. (2020)
dCas13b-CIBN CRY2PHR-FTO	Effect photoactivatable RNA m^6^A level reduction	*MALAT1*, *Alp*, *Bglap*, *RunX2*, and *Sp7, Pth1r*	Zhao et al. (2020)
dCas13b-ALKBH5	Demethylates m^6^A of targeted mRNAs to enhance their stability	*CYB5A, CTNNB1, EGFR, MYC*	Li et al. (2020)
**Modulating m^6^A reading**			
dCas13b-YTHDF2	Induces RNA degradation	*KRAS, PPIB*	Rauch et al. (2018)
dCas13b-YTHDF1	Enhances translation with minimal mRNA destabilization effect	Firefly luciferase	Rauch et al. (2018)

*CRISPR, clustered regularly interspaced short palindromic repeats; m^6^A, N^6^-methyladenosine.*

### Restoring Abnormal Loss of N^6^-Methyladenosine

Gene transcripts that have lost m^6^A because of malfunction of the methyltransferases (METTL3 and/or METTL14) in the m^6^A methylation complex can be repaired using the CRISPR–dCas13 system. This is achievable by fusing dCas13, localized in the nucleus or cytoplasm, with a methyltransferase domain-truncated METTL3 or a modified METTL3:METTL14 complex, respectively. The resultant CRISPR–dCas13 constructs were able to install m^6^A marks, in a site-specific manner, on hypomethylated mRNAs or mRNAs with amendable m^6^A levels, including *Sox2*, *Foxm1*, and *Znf638* in human cells (Wilson et al., 2020). Light-mediated m^6^A editing has also been put forward as another appealing CRISPR–dCas13 system for engineering the m^6^A methylome and worth close examination for applicability of the principle in therapeutics. The photoactivatable m^6^A editing CRISPR–dCas13b tool, which has a coupled component made of the methyltransferase domains of METTL3 and METTL14, was effectively employed in adding m^6^A to gene transcripts (*TPT1, ACTB, TUG1*) in human cells (Zhao et al., 2020).

### Correcting N^6^-Methyladenosine Removal Incompetence

In the context of m^6^A erasure (demethylation), the m^6^A demethylase FTO and ALKBH5 can be incorporated into the CRISPR–dCas13 system to effect targeted removal of m^6^A on hypermethylated mRNAs or induce hypomethylation as corrective measures. By utilizing a CRISPR–dCas13b–FTO construct, Mo et al. (2020) were able to make mRNAs more stable *via* site-directed demethylation (Table 3) (Mo et al., 2020). Similarly, by applying a photoactivatable FTO-coupled CRISPR–dCas13b strategy, m^6^A marks were effectively and in a targeted manner removed on endogenous gene transcripts (Zhao et al., 2020). Methylated mRNAs that are preferentially demethylated by ALKBH5 can also be targeted by a CRISPR–dCas13b–ALKBH5 construct to remodel their m^6^A milieu, as applied in reducing the m^6^A levels associated with transcripts like *CYB5A, CTNNB1, EGFR*, and *MYC*, leading to their increased stability and translation (Li et al., 2020).

### Rescuing Defective N^6^-Methyladenosine Reading

It is also possible to specifically target m^6^A readers to mRNAs of interest using the CRISPR–dCas13b system. It also implies that m^6^A binding protein dysfunctionality due to mutation can be rectified with a CRISPR–dCas13b construct fused to an engineered functional version of the relevant defective m^6^A reader. For example, YTHDF1 and/or YTHDF2, the two well-characterized m^6^A readers, can be fused to CRISPR–dCas13b and guided to specific mRNAs for m^6^A reading and subsequent fate alteration. Both CRISPR–dCas13b–YTHDF1 and CRISPR–dCas13b–YTHDF2 constructs were able to effect the native functions of YTHDF1 and YTHDF2, leading to translation enhancement and mRNA degradation in cells, respectively (Rauch et al., 2018).

### Targeted N^6^-Methyladenosine Editing in the Diseased Brain as a Promising Treatment Strategy

Based on the intriguing outcomes and specificity of m^6^A editing application *in vitro* (Table 3 and Figure 4), we hereby propose the CRISPR–dCas13 system as a highly efficient tool for precise targeting and repair of aberrant m^6^A-modified mRNAs implicated in the pathophysiology of pertinent neurological disorders. Such a tool can be potentially useful in treating neurological disorders known to have pathologic m^6^A mRNA methylation, demethylation, or reading as the central underlying pathogenesis mechanism (Table 2). Employing high-resolution single-nucleotide binding techniques will be critical for identifying specific nucleotides bearing the abnormal m^6^A modification in the diseased brain. This will improve targeting, leading to the desired effect. An example of a strategy for improving the identification and targeting of nucleotides harboring disease-causing m^6^A marks is by adopting the enhanced crosslinking and immunoprecipitation (eCLIP) technique for robust factor-specific profiling of the m^6^A methylome in the pathologic brain (Van Nostrand et al., 2016). The *in vivo* experimental approach for investigating the potency of the CRISPR–dCas13 system for resolving neurological disorders caused by m^6^A dysregulation would include modeling the disorder in experimental animals and treating them with the rescuing CRISPR–dCas13 construct(s) that would have a target effect in the brain and on the implicated pathogenic mRNA (Figure 4). While the idea of investigating the application of the CRISPR–dCas13 system for rectifying aberrant m^6^A mRNA methylation (Figure 4) implied in neurological disorders sounds interesting, the approach may be fraught with challenges, especially in preventing off-target effects and in rescuing phenotypes of complex syndromic neurological disorders (e.g., ASD, AD, schizophrenia, MDD). Further investigations that can reveal convergent downstream effectors underlying the pathophysiology of polygenic neurological disorders caused by defective m^6^A signaling can help streamline an m^6^A editing-mediated therapeutic strategy.

## Conclusion

Methylation of mRNA has emerged as a posttranscriptional regulation of gene expression that modulates protein synthesis in cells. Studies have shown that the m^6^A mRNA methylation machinery, composed of writers, erasers, and readers (Figure 1A), critically and extensively regulates RNA metabolism (trafficking, stability, processing, and translation efficiency) in cells to impact major biological processes. The brain is a hub of m^6^A modification, and the enrichment of m^6^A in the brain is reflective of the essential role it plays in optimal brain morphogenesis and functionality. Hence, the m^6^A interactome is known to regulate several neurodevelopmental processes in the brain, including neurogenesis, gliogenesis, synapse formation, and neuronal activity. Many of the m^6^A factors appear to have multiple functional effects during cortical development and in orchestrating several aspects of brain physiology (Table 1). This may make it challenging to effectively disentangle the rather multifactorial downstream causatives or complex phenotypic effects elicited by the dysregulation of the m^6^A machinery in the brain. As a typical example, whereas the *FTO rs9939609* A variant is a risk factor for brain atrophy in old age (Ho et al., 2010) and AD development (Keller et al., 2011), it appears to be neuroprotective against MDD (Samaan et al., 2013). It also implies that m^6^A signaling is worth considering as a pivotal pathway that can cause novel syndromic neurological disturbances. Indeed, some inherited or acquired defects in the m^6^A RNA methylome are known causes of syndromes such as ASD, Smith–Magenis syndrome, and FXS. It also goes to reason that genetic variants of factors that make the m^6^A machinery pose a risk for certain (novel) non-syndromic neurological anomalies of the brain.

The phenomenal neurodevelopmental role played by m^6^A mRNA methylation and implication for neurological perturbations provoke considerable attention to the emerging involvement of the m^6^A methylome in normal brain structure and function maintenance. Going forward, more robust and advanced probing techniques are required to finely dissect the mechanistic basis of m^6^A-mediated neurodevelopment and its involvement in the pathophysiology of pertinent neurological disorders of the brain. Such sophisticated investigations may uncover therapeutic cues that can potentially fend off the neurological disorders caused by defective RNA methylation in the brain or alleviate the associated symptoms. For now, the application of the CRISPR–dCas13 system to edit m^6^A to restore normality of mRNA state and fate in, say, brain disease conditions is one of the promising approaches for treating abnormal m^6^A signaling-related neurological disorders.

## Author Contributions

GS, YX, HN, and TT all contributed to writing and editing the manuscript. All authors contributed to the article and approved the submitted version.

## Conflict of Interest

The authors declare that the research was conducted in the absence of any commercial or financial relationships that could be construed as a potential conflict of interest.
